# Interactions of zebrafish peptide YYb with the neuropeptide Y-family receptors Y4, Y7, Y8a, and Y8b

**DOI:** 10.3389/fnins.2013.00029

**Published:** 2013-03-15

**Authors:** Görel Sundström, Tomas A. Larsson, Bo Xu, Johan Heldin, Dan Larhammar

**Affiliations:** Department of Neuroscience, Uppsala UniversityUppsala, Sweden

**Keywords:** NPY, PYY, G-protein-coupled receptor, zebrafish, evolution

## Abstract

The neuropeptide Y (NPY) system influences numerous physiological functions including feeding behavior, endocrine regulation, and cardiovascular regulation. In jawed vertebrates it consists of 3–4 peptides and 4–7 receptors. Teleost fishes have unique duplicates of NPY and PYY as well as the Y8 receptor. In the zebrafish, the NPY system consists of the peptides NPYa, PYYa, and PYYb (NPYb appears to have been lost) and at least seven NPY receptors: Y1, Y2, Y2-2, Y4, Y7, Y8a, and Y8b. Previously PYYb binding has been reported for Y2 and Y2-2. To search for peptide-receptor preferences, we have investigated PYYb binding to four of the remaining receptors and compared with NPYa and PYYa. Taken together, the most striking observations are that PYYa displays reduced affinity for Y2 (3 nM) compared to the other peptides and receptors and that all three peptides have higher affinity for Y4 (0.028–0.034 nM) than for the other five receptors. The strongest peptide preference by any receptor selectivity is the one previously reported for PYYb by the Y2 receptor, as compared to NPY and PYYa. These affinity differences may be helpful to elucidate specific details of peptide-receptor interactions. Also, we have investigated the level of mRNA expression in different organs using qPCR. All peptides and receptors have higher expression in heart, kidney, and brain. These quantitative aspects on receptor affinities and mRNA distribution help provide a more complete picture of the NPY system.

## Introduction

The neuropeptide Y (NPY) system of receptors and peptides in vertebrates is complex with 4–7 G-protein coupled receptors and 3–4 peptide ligands depending on the species. Members of both the receptor and peptide families have been found in invertebrates as well as vertebrates, suggesting an ancient origin of the system (Tensen et al., 1998; Garczynski et al., 2002; Hill et al., 2002; Larhammar and Salaneck, 2004). The receptor family expanded in the two whole genome duplications (tetraploidizations) early in vertebrate evolution, resulting in a repertoire of seven receptors named Y1 through Y8 in a hypothetical ancestral gnathostome (Fredriksson et al., 2004). (Receptor Y3 is missing; this receptor was postulated from pharmacological studies but remains unidentified as a separate gene.) The full repertoire is still present in a cartilaginous fish, the elephant shark, *Callorhinchus milii* (Larsson et al., 2009). At the time of the teleost-specific whole genome duplication the teleost ancestor seems to have had a repertoire of five receptors (Salaneck et al., 2008). After the teleost-specific tetraploidization only the new copy of Y8 was retained, all other duplicates seem to have been lost.

The receptors can be divided into three subfamilies, the Y1, Y2, and Y5 subfamilies. Teleost fishes have 3–4 members in the Y1 subfamily depending on species, Y1, Y4 (formerly called Ya), Y8a (Yc), and Y8b (Yb). Teleosts have the same repertoire as amphibians and birds in the Y2 subfamily, Y2 and Y7, whereas mammals have lost the latter. No teleost has yet been found to possess a Y5 receptor, suggesting that this was lost in the common ancestor of the teleost lineage (Salaneck et al., 2008; Larsson et al., 2009). We recently described a local duplicate of the Y2 receptor in zebrafish and medaka, called Y2-2 (Fallmar et al., 2011). Bioinformatic studies have shown that the teleost tetraploidization resulted in four peptides, NPYa, NPYb, PYYa, and PYYb (formerly called PY). Medaka (*Oryzias latipes*) seems to have lost PYYb and zebrafish (*Danio rerio*) lacks NPYb (unless they have escaped sequencing in the genome projects), while other species with sequenced genomes have the full repertoire of four peptides: green spotted pufferfish (*Tetraodon nigroviridis)*, fugu (*Takifugu rubripes)*, and three-spined stickleback (*Gasterosteus aculeatus*) (Larsson et al., 2009).

The NPY system has attracted considerable attention during the past several years due to its function on appetite regulation. In mammals the system is able to both enhance and decrease food intake depending on which receptor-peptide combination is activated (Suzuki et al., 2010; Zhang et al., 2011). Other physiological processes that can be influenced by NPY-family peptides, as mostly studied in mammals, are blood pressure, anxiety, regulation of pituitary release of hormones, bone formation, and pain (Pedrazzini et al., 2003; Lee and Herzog, 2009; Zhang et al., 2011). Until recently, neither of the receptors that stimulate appetite in mammals, Y1 and Y5, had been found in fishes. This was puzzling because feeding studies in goldfish have shown that administration of NPY enhances feeding (Narnaware et al., 2000; Narnaware and Peter, 2001). The zebrafish Y1 receptor has been identified using whole genome data (Salaneck et al., 2008; Larsson et al., 2009). Its single intron has expanded to approximately 40 kb which partially explains why the receptor was not found by PCR. Interestingly, the receptor was not present in any of the other four fully sequenced teleost genomes available (Salaneck et al., 2008; Larsson et al., 2009), but it has been cloned in more basal teleosts such as bowfin (*Amia calva*) and Atlantic herring (*Clupea harengus*) (Salaneck et al., 2008).

Functional and evolutionary studies in mammals have shown that their Y4 receptor has evolved a partnership with pancreatic polypeptide (PP). It should be stressed that PYYb is not the teleost fish ortholog of PP, instead the latter is a tandem duplicate of PYY that probably took place early in the tetrapod lineage. The selectivity of PP for Y4 is particularly strong in rat and mouse. In contrast, chicken PP, PYY, and NPY bind to Y4 with equal affinities. Thus, the PP-Y4 preference seems to have evolved in mammals (Lundell et al., 1995, 2002). One aim of the present study was to investigate if similar partnerships have arisen between NPY-family peptides and receptors in zebrafish.

Pharmacological characterization of the Y2, Y2-2, Y4, Y7, Y8a, and Y8b receptors in zebrafish, using the endogenous ligands NPY (i.e., NPYa) and PYYa, has previously been reported from our laboratory (Lundell et al., 1997; Ringvall et al., 1997; Starback et al., 1999; Berglund et al., 2000; Fredriksson et al., 2004, 2006). After PYYb was discovered, it was included in the characterization of Y2 and Y2-2 (Fredriksson et al., 2006; Fallmar et al., 2011; Zhang et al., 2011). The zebrafish Y1 receptor will be described in detail separately (Larson and Larhammar, in preparation). What is missing is a description of PYYb binding to receptors Y4, Y7, Y8a, and Y8b which is therefore the focus of the present study. We also report the mRNA distribution of the NPY-family peptides and receptors as determined by qPCR.

## Materials and methods

### Cloning and transfection

The zebrafish receptors have previously been cloned and expressed in cell lines by our laboratory. The zebrafish Y8a and Y8b receptors were inserted in a pTEJ-8 vector and stably expressed in CHO-cells (Ringvall et al., 1997; Berglund et al., 2000). Y7 has been inserted in a pCEP4 vector and semi-stably expressed in HEK 293-EBNA cells (Fredriksson et al., 2004). The zebrafish Y4 receptor coding region was inserted in a pTEJ-8 vector (Starback et al., 1999) and transient expressed in HEK cells. The cell-lines were grown according to the recommendations of the manufacturer. Ninety percent of the confluent cells were harvested and mixed with 100 μl 25 mM HEPES buffer (pH 7.4 and 2.5 mM CaCl_2_ and 1 mM MgCl_2_) and stored at −80°C.

### Binding assay

Thawed aliquots of transfected cells were mixed with 1 ml 25 mM HEPES buffer (pH 7.4, 2.5 mM CaCl_2_, 1 mM MgCl_2_ and 0.2 g/l bacitracin). The samples were homogenized and another 1 ml buffer was added. ^125^I-pPYY was used as radioligand in all experiments, radioligand, and unlabeled ligands (serial concentrations of zfNPY, zfPYYa, and zfPYYb respectively) and cells were mixed at the proportion 2:1:1 in 100 μl reactions followed by 2 h incubation. The zebrafish NPY (zfNPY) and PYYa (zfPYYa) were synthesized at Eli Lilly and Company. The zebrafish PYYb (zfPYYb) was supplied in a crude form by GL Biochem Shanghai and purified as previously described (Fredriksson et al., 2006). The incubation was terminated by rapid filtration through GF/C filters pre-soaked in 0.3% polyethyleneimin, using a TOMTEC cell harvester (Orange, CT, USA). The filters were washed with 50 mM Tris-HCl (pH 7.4) and dried at 50°C for 30 min. Filters were treated with MeltiLex A melt on scintillator sheets (Perkin Elmer, Boston, MA, USA). The radioactivity was measured using a Wallac 1450 Betaplate counter and the results were analyzed using Prism 4.0 software package (GraphPad, San Diego, CA, USA). The pK_i_ values were compared to each other using One-Way ANOVA followed by Tukey-Kramer Multiple Comparison Test (GraphPad, San Diego, CA, USA).

### RNA isolation and cDNA synthesis

Four adult zebrafish were purchased from Akvarie Hobby (Uppsala, Sweden) and all procedures involving animals were conducted in accordance with approval #C264/6 from the local ethics committee. The animals had been kept on a regular daily feeding scheme. The following tissues were obtained by dissection: eye, muscle, heart, spleen, GI, liver, kidney, and brain. The tissues were frozen at −80°C until the preparation of total RNA. Total RNA was prepared using the Trizol Reagent (Invitrogen, Sweden) following the manufacturer's instructions. cDNA was synthesized using M-MLV reverse transcriptase (Invitrogen, Sweden) and random hexamers as primers following the manufacturer's recommendations. Absence of genomic DNA in the cDNA was controlled for by PCR.

### Primer design and quantitative real-time PCR

Primers for the quantitative PCR were designed using the Beacon Designer v4.0 (Premier Biosoft, USA). The primer sequences are listed in Table 1. The optimal annealing temperatures for each primer combination were determined by PCR on genomic DNA. All real-time PCR reaction had a total volume of 20 μl and contained cDNA synthesized from 5 ng of total RNA, 5 pM of each primer, 0.64 mM MgCl_2_, 0.2 mM dNTP, SYBR Green (1:50 000), 0.02 u/μl Taq DNA polymerase and buffer (without MgCl_2_) (Invitrogen, Sweden). The reactions were run on a MyiQ thermal cycler (Bio-Rad Laboratories, Sweden) under following conditions: 95°C for 4 min, followed by 50 cycles at 95°C for 15 s, 59°C for 30 s, and 72°C for 30 s. To confirm that only one product was formed, a melt curve analysis with 84 cycles at 55°C for 10 s, increased by 0.5°C per cycle were conducted.

**Table 1:** **The primer sequences used for the quantitative PCR**.

	**Sense primer**	**Anti-sense primer**
zfNPY	CTTGTTCGTCTGCTTGG	GTGCTGAATAATACTTGGC
zfPYYa	TCTGCGTGCTTCTGTGTC	CGTGATGAGGTTGATGTAGTG
zfPYYb	TCTGTGCGTTATAGTGTG	TGATGTAGTGTCTTAGAGC
zfY2	TAGTTGTCATCGCCATC	CCATCTGTGCTACTTCC
zfY4	TGCTGTCCTCTGCTCCTC	CGCCTGTGTTCATCCTCTC
zfY7	GCAGTATGTGGTCCCTTTG	CATCTTGGTGGTCTTCTTCC
zfY8a	TCTCATTGGTGCTCATTGC	GGATGTTGAAGGATAGGAAGG
zfY8b	GCGGAGGACGACAGAAG	GGGAGCCAACACAAAGC

### Data analyses and calculation of relative expression levels

The data were analyzed and threshold cycle (Ct) values derived using MyIQ software v 1.04 (Bio-Rad Laboratories, Sweden). Differences in the primer efficiency were corrected for using LinRegPCR (Ramakers et al., 2003), Grubbs test for outliers (GraphPad, USA) was used to calculate the mean primer efficiency for each primer pair and detection and exclusion of outliers. The Ct values were transformed into quantities using the delta Ct method (Livak and Schmittgen, 2001) and the highest expression was set to 1. geNorm (Vandesompele et al., 2002) was used to identify the most stable housekeeping gene which was thereafter used to normalize expression due to differences in cDNA concentration between samples.

## Results

### Binding assay

**Figure 1:**
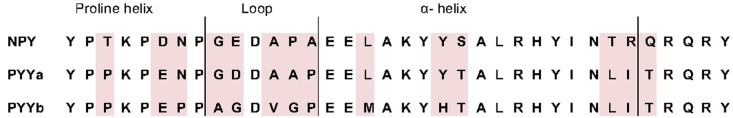
**Alignment of the mature peptides in the NPY family in zebrafish.** Amino acid differences are shadowed. Proposed tertiary structures are described at the top.

The zebrafish receptors were expressed in mammalian cells and the membrane fraction was used for binding experiments. Competition binding experiments were performed using the three endogenous ligands NPY, PYYa and PYYb in competition with iodinated porcine PYY. The mature 36 amino acid PYYb peptide has fourteen differences compared to NPY and seven when compared with PYYa (see Figure 1). PYYa differs from NPY at nine positions. Thus, PYYb has clearly changed more than PYYa after duplication of their common ancestral gene.

**Figure 2:**
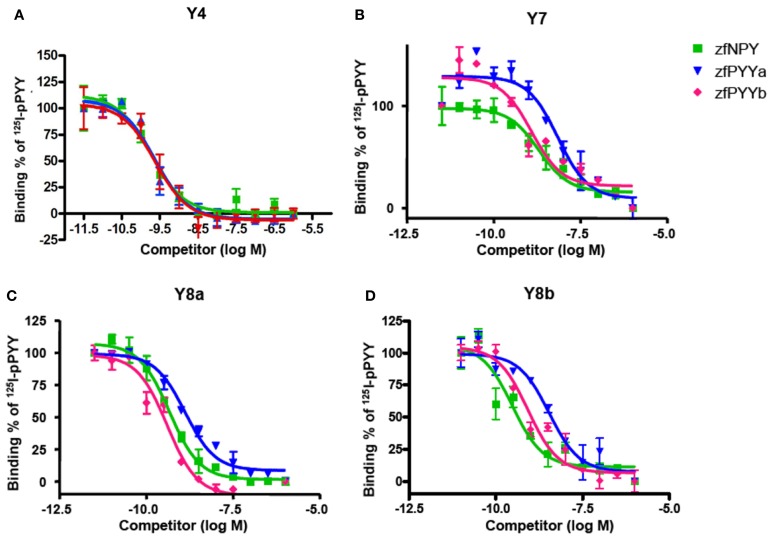
**Binding curves for each receptor.** Panels **(A–D)** are the representative binding curves for Y4, Y7, Y8a and Y8b, respectively. The *x*-axis is the logarithmic concentration of unlabeled ligand. All experiments were done in duplicate. Note that the curve shown for the binding of zfPYYa to Y8b displays greater difference to the other peptides than if all experiments are considered (see Table 2).

In Table 2, the equilibrium inhibition constants (*K*_*i*_) for Y4, Y7, Y8a, and Y8b are listed and representative inhibition curves are shown in Figure 2. The affinity of PYYb for the Y7 receptor was almost identical to that of NPY (0.59 and 0.57 nM) whereas PYYa has a significantly lower affinity, 1.82 nM. No difference in affinity was detected between ligands tested on the Y4, Y8a, and Y8b receptor.

**Table 2:** **The *K*_*i*_ values and standard error of the mean for four zebrafish receptors and their endogenous ligands**.

	**Y4(Ya)**		**Y7**		**Y8a (Yc)**		**Y8b (Yb)**	
**Ligand**	***K*_*i*_ (nM) ± *SEM***	***n***	***K*_*i*_ (nM) ± *SEM***	***n***	***K*_*i*_ (nM) ± *SEM***	***n***	***K*_*i*_ (nM) ± *SEM***	***n***
zfNPY	0.033 ± 0.005	3	0.57 ± 0.16	5	0.21 ± 0.07	7	0.13 ± 0.04	4
zfPYYa	0.034 ± 0.009	3	1.8 ± 0.14	3	0.51 ± 0.001	6	0.25 ± 0.12	3
zfPYYb	0.028 ± 0.013	3	0.59 ± 0.17	5	0.32 ± 0.15	4	0.22 ± 0.14	4

n, number of experiments done. The Kd value of pPYY for the Y7 receptor is from Fredriksson et al. (2004), for Y4 from Starback et al. (1999) and for Y8a and Y8b receptor from Berglund et al. (2000).

### Expression levels for mRNA

**Figure 3:**
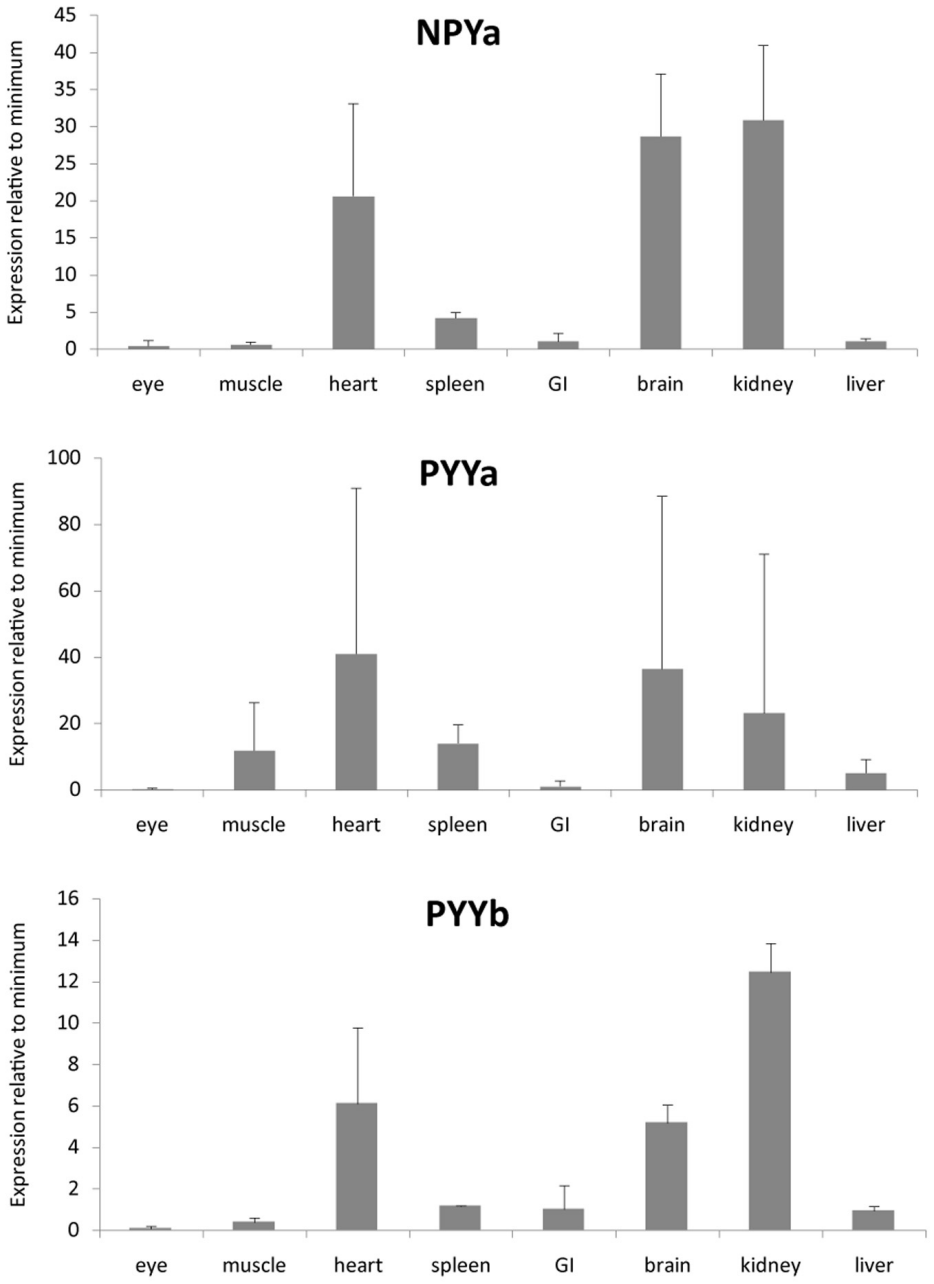
**Expression data for neuropeptide Y peptides in a panel of eight zebrafish organs.** Error bars display the standard error of the mean. Normalized Ct values were used to calculate the relative expression values. For each transcript the tissue with the lowest expression was used to calculate relative expression. For all genes, the eye had lowest expression. The analysis was performed twice, each time with duplicate samples.

**Figure 4:**
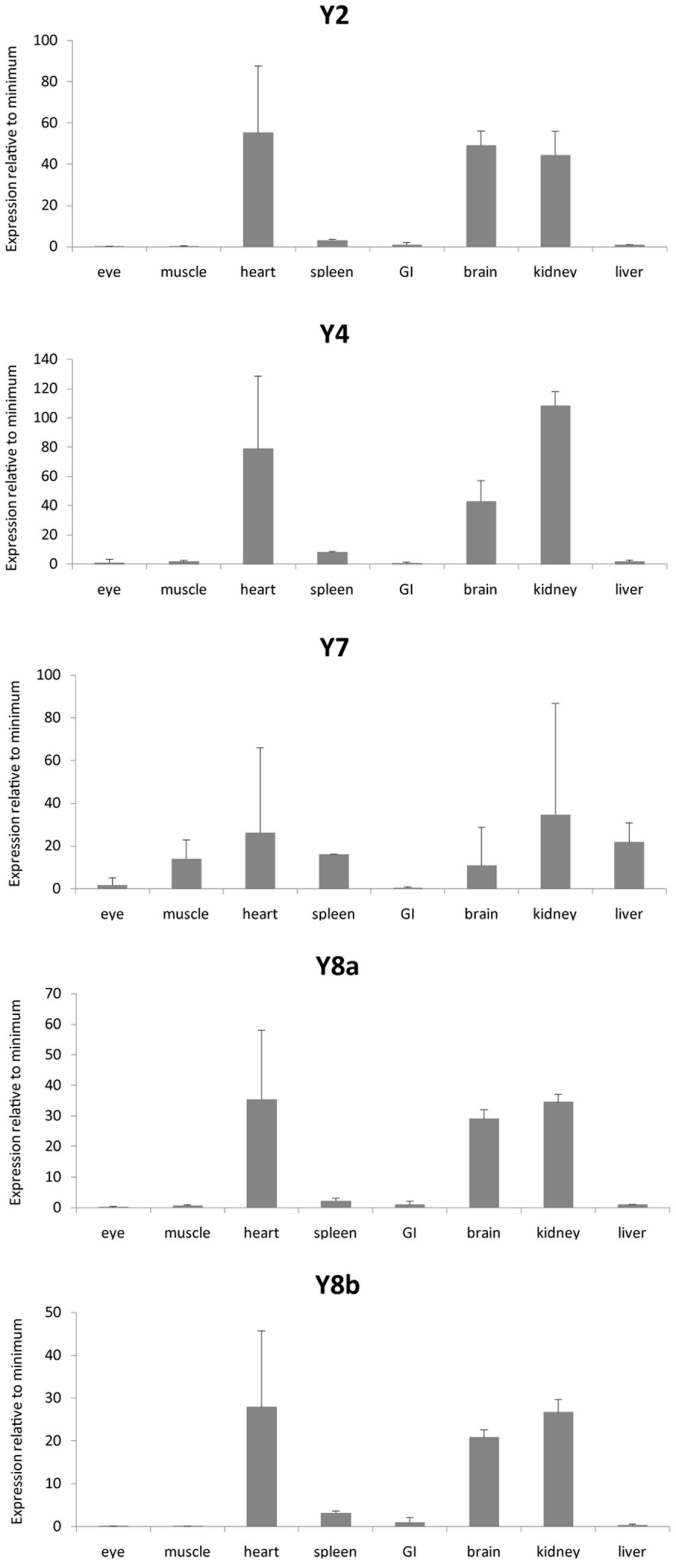
**Expression data for neuropeptide Y receptors in a panel of eight zebrafish organs.** Error bars display standard error of the mean. Normalized Ct values were used to calculate the relative expression values. For each transcript the tissue with the lowest expression was used to calculate relative expression. For the different genes, the following tissues had lower expression: Y2, eye; Y4, GI; Y7, GI; Y8a, eye; Y8b, muscle. The analysis was performed twice, each time with duplicate samples.

We investigated the mRNA expression level in a panel of eight organs (Figure 3). Ef1-α was used to normalize expression due to differences in cDNA concentration between samples for all tissues. All three peptides show a similar expression pattern with high expression in kidney, heart, and brain. PYYb was the only peptide with clear expression in the gastrointestinal tract (GI). Note that the scales on the Y axes differ. The receptors too have expression patterns that resemble one another with the main mRNA expression in heart, kidney, and brain (Figure 4). The Y7 receptor also has expression in liver.

## Discussion

The main objective of this study was to examine the affinity of PYYb for the Y4, Y7, Y8a, and Y8b receptors, and to compare it to the other two endogenous peptides, NPY and PYYa. Before this study, PYYb had only been analyzed with regard to its binding to the Y2 and Y2-2 receptors with the result that Y2 displayed a clear preference for PYYb, the difference in affinity in comparison with PYYa being as great as 50-fold (Fredriksson et al., 2006).

In our present study, PYYb performs most similar to NPY despite the larger number of sequence differences between these two peptides (14) than between PYYb and PYYa (7), as well as PYYa and NPY (9). The most deviating binding constants are noted for PYYa which has a 3-fold lower affinity than NPY and PYYb for the Y7 receptor and slightly lower than the other two peptides for Y8a. The zebrafish Y7 receptor is 50% identical to zebrafish Y2 and PYYa has low affinity for both of these. This observation will be interesting to correlate with the physiological roles of the peptides and receptors in zebrafish. No differences in affinities were detected for the three zebrafish peptides in previous studies of the rainbow trout (*Onchorhynchus mykiss*) Y7 receptor. Zebrafish Y7 and rainbow trout Y7 have an amino acid identity of approximately 73% (Larsson et al., 2006). None of the receptors studied here showed the same kind of ligand preference as the Y2 receptor (Fredriksson et al., 2006). This indicates that Y2 may have a unique ligand partnership with PYYb in zebrafish, just as in the case for Y4 and PP in mammals. The evolutionary distance between zebrafish and trout has been estimated to be more than 300 million years (Hedges et al., 2006 http://www.timetree.org/), so there has been ample time to evolve differences in binding preference. The differences in affinity, comparing both within and between species, will be useful for precise mapping of the points of interaction between peptide ligands and receptor in three-dimensional modeling.

PYYa and PYYb are the result of a whole genome duplication that took place early in teleost evolution more the 300 million years ago (Sundstrom et al., 2008). All peptides in the family are believed to have a tertiary structure called the PP-fold or hairpin structure. The first eight amino acids are involved in a proline-helix followed by a β-turn constructed of position number 9–14 (see Figure 1). The remaining positions form an alpha helix, except for the last five (Darbon et al., 1992; Keire et al., 2000). Only two amino acids differ between PYYa and PYYb in the alpha-helix but four out of six positions have changes in the β-turn region (Figure 1). Only one amino acid has been changed in the proline helix but the change is from an aspargine to a proline which might have implications for the tertiary structure. These changes could possibly influence the binding pattern, even if the highly conserved amidated C-terminal of the peptide has been found to be the part most involved in receptor binding. The loop region has not previously been shown to have any effect on binding to the different Y receptors (Berglund et al., 2000).

Studies of how NPY affects appetite have been conducted in several fish species (Narnaware et al., 2000; Narnaware and Peter, 2001) and the NPY system seems to influence food intake, although the orexigenic receptor subtypes have not been identified in the fish species studied. The receptors that in mammals are involved in inhibition of appetite have been known in zebrafish for several years and there have been some studies investigating how PYY can inhibit feeding. In mammals it seems like the main inhibitory action on appetite is performed by PYY3-36. This peptide is the result of cleavage of the two first amino acids by dipeptidyl peptidase IV (Mentlein et al., 1993). This processing probably turned out to be favorable because it results in selectivity for Y2 over the other receptor subtypes, of which especially Y1 is expressed in blood vessels, at least in mammals. However, all known non-mammalian PYY peptides start with the sequence Tyr-Pro-Pro (Cerda-Reverter and Larhammar, 2000; Sundstrom et al., 2008) and the peptidase is unable to cleave between two proline residues. Thus, non-mammalian PYY cannot be processed to the Y2-preferring PYY3-36 in non-mammals. However, the non-selectivity of intact PYY may have no biological consequences if no other receptor subtypes can be targeted by PYY circulating in the blood.

Measurements of the changes of PYYa expression in fed and fasted fishes have been conducted for several species. No expression difference was detected in either brain or GI tract for salmon (Murashita et al., 2009), while fed goldfish showed a higher expression of PYYa in the brain compared to the unfed (Gonzalez and Unniappan, 2010). One study has also investigated the expression pattern of PYYb in yellowtail (*Seriola quinqueradiata*) showing a higher expression in the intestine in fasted fishes compared to fed fishes (Murashita et al., 2006). Goldfish have been injected with full-length PYYa either intraperitoneally or intracerebroventricularly. Independent of administration method, food intake was reduced by approximately 30% (Gonzalez and Unniappan, 2010). Both the salmon lineage and the goldfish/carp lineage have however undergone additional separate whole genome duplications (i.e., a fourth tetraploidization) which might have resulted in extra receptors (and peptides) in the NPY system.

PYY and PP are mainly expressed in endocrine cells in the GI in mammals (Larhammar and Salaneck, 2004). Zebrafish has two different PYY peptides and in our quantitative PCR analysis it was possible to detect some difference in the tissue distribution between PYYa and PYYb. PYYb has some expression in the GI (Figure 3) which indicates that subfunctionalization has occurred since the duplication. Neither the peptides nor the receptors showed any expression in the eye. Among the receptors, Y7 was the only one investigated that showed expression in liver. The NPY system seems to be prominent in the kidney because all three peptides and all five receptors were abundant there, suggesting perhaps a role in electrolyte regulation. These findings encourage detailed studies with *in situ* hybridization to see which cell types express the peptides and the receptors.

Zebrafish diverged early in teleost evolution and it is the only fully sequenced teleost where a Y1 receptor has been described. Studies of the binding to the Y1 receptor show similar affinities for all three peptides (Larson and Larhammar, in preparation). Taken together, these results show that some lineage specific peptide-receptor affinity differences have evolved in the teleost lineage after duplication of the NPY system genes. Y2 may have a unique ligand partnership with PYYb and the PYYa peptide has low affinity for both Y7 and Y2. Functional studies are required to see if the affinity differences have physiological importance. Some subfunctionalization regarding tissue distribution seems also to have occurred both among the peptides and receptors. These comprise important aspects to take into consideration to understand the complex NPY system of teleost fish.

### Conflict of interest statement

The authors declare that the research was conducted in the absence of any commercial or financial relationships that could be construed as a potential conflict of interest.
